# Supplementary material for: Impact of GSTA1 Polymorphisms on Busulfan Oral Clearance in Adult Patients Undergoing Hematopoietic Stem Cell Transplantation
Source: Pharmaceutics. 2019 Sep 1;11(9):440. doi: 10.3390/pharmaceutics11090440 (PMC6781287; doi:10.3390/pharmaceutics11090440)
Supplement: Supplementary file 1 [file pharmaceutics-11-00440-s001.pdf]

pharmaceutics

Supplementary Materials: Impact of *GSTA1* Polymorphisms on Busulfan Oral Clearance in Adult Patients Undergoing Hematopoietic Stem Cell Transplantation

Veronique Michaud, My Tran, Benoit Pronovost, Philippe Bouchard, Sarah Bilodeau, Karine Alain, Barbara Vadnais, Martin Franco, François Bélanger and Jacques Turgeon

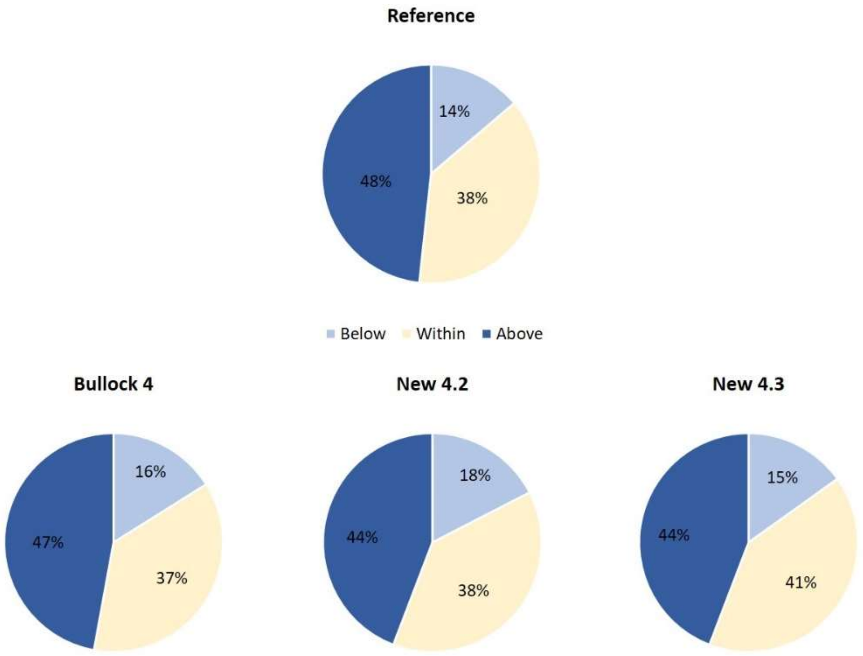

Figure S1. Percentage of patients with an AUC below, within and above the therapeutic range after the initial oral dose of busulfan using the refence AUC vs. 3 limited sampling methods (LSMs) based on 4 blood samples: Bullock 4, New 4.2 and New 4.3.
